# Molecular Survey of *Toxoplasma gondii* in Wild Mammals of Southern Italy

**DOI:** 10.3390/pathogens12030471

**Published:** 2023-03-16

**Authors:** Hiba Dakroub, Giovanni Sgroi, Nicola D’Alessio, Danilo Russo, Francesco Serra, Vincenzo Veneziano, Simona Rea, Alessia Pucciarelli, Maria Gabriella Lucibelli, Esterina De Carlo, Giovanna Fusco, Maria Grazia Amoroso

**Affiliations:** 1 Laboratory of Animal Ecology and Conservation (AnEcoEvo), Department of Agriculture, University of Naples Federico II, 80055 Portici, Italy; 2 Department of Animal Health, Istituto Zooprofilattico Sperimentale del Mezzogiorno, 80055 Portici, Italy; 3 Osservatorio Faunistico Venatorio of Campania Region, Istituto Zooprofilattico Sperimentale del Mezzogiorno, 80055 Portici, Italy; 4 Department of Veterinary Medicine, University of Naples Federico II, 80137 Naples, Italy

**Keywords:** parasite, prevalence, apicomplexan, *Canis lupus*, *Meles meles*, *Capreolus capreolus*, *Sus scrofa*, *Vulpes vulpes*, zoonosis

## Abstract

Systematic wildlife surveillance is important to aid the prevention of zoonotic infections that jeopardize human health and undermine biodiversity. *Toxoplasma gondii* is an opportunistic zoonotic protozoan that can infect all endothermic vertebrates, causing severe disease in immunocompromised humans and cases of congenital transmission. Humans can be infected by ingestion of raw meat containing bradyzoites or water contaminated by oocysts. In our study, we assessed the potential circulation of *Toxoplasma gondii* in wild mammals by performing surveillance in the Campania region (southern Italy) and surveyed its presence from 2020 to 2022 within the framework of the Regional Plans for Wildlife Surveillance. In detail, 211 individuals belonging to five wild mammals (wolf, fox, wild boar, badger, and roe deer) underwent necropsy and the organs were analyzed by real-time PCR for the detection of the parasite. *Toxoplasma gondii* was found in 21.8% (46/211) of the subjects examined. No statistically significant differences were noticed between the prevalence and the host’s trophic level or age, rejecting the hypotheses that *Toxoplasma gondii* will have a higher prevalence in top predators and adult individuals, respectively. Our work emphasized the high circulation of *Toxoplasma gondii* in wildlife and remarked on the critical role of anthropized areas where domestic cats and wildlife may come into contact, urging a systematic surveillance.

## 1. Introduction

*Toxoplasma gondii* (Conoidasida, Sarcocystidae) is an apicomplexan protozoan that causes a zoonotic infection known as toxoplasmosis. This parasite is one of the most resilient and persistent living parasites, able to infect many endothermic vertebrates including humans [1]. The Food and Agriculture Organization of the United Nations (FAO) identified toxoplasmosis among the 10 most important foodborne diseases [2]. The definitive hosts of *Toxoplasma gondii* are wild and domestic felids, since these animals facilitate the parasite’s sexual recombination and shed millions of stable unsporulated oocysts into the environment through their faeces [3]. Yet, the intermediate host range of *Toxoplasma gondii* is incredibly broad, including humans, domestic animals, and wild vertebrates [4]. These intermediate hosts support the asexual forms of tachyzoite and bradyzoite tissue cysts of the parasite, which invade the host’s small intestine after consumption [3].

Humans can be infected via different ways: (1) by eating undercooked meat of animals harbouring bradyzoites in the tissue cysts; (2) by ingesting food or water contaminated with oocysts shed with faeces; (3) by blood transfusion or organ transplantation; and (4) through the placenta, from mother to the foetus with tachyzoites [5,6].

During the initial infection phase of an intermediate host, comprising humans, *Toxoplasma gondii* replicates rapidly and spreads throughout the tissues, including the brain (acute toxoplasmosis). Eventually, parasite replication slows down, and the protozoa cluster together in tissue cysts (latent toxoplasmosis). Humans with latent toxoplasmosis who become immunocompromised may develop reactivated toxoplasmosis, in which the dormant parasites in the tissue cysts will start replicating again. This reactivation can cause severe flu-like symptoms, blurred vision, or toxoplasmic encephalitis [7]. Recently, many epidemiological studies associated latent toxoplasmosis with a wide variety of cognitive and neuropsychiatric disorders including Alzheimer’s, bipolar disorders, epilepsy, obsessive compulsive disorders, and schizophrenia [8]. This association seems to be determined by the interference between the parasite and the expression of many neurotransmitters [8].

Despite this new and important evidence and considering that the parasite is estimated to persist chronically in about 30% of the human population, toxoplasmosis is underestimated in terms of monitoring, preventing, and treating it [9]. Studies that contribute to a better understanding of the pathogen’s distribution and prevalence, as well as the factors that influence its prevalence, are, therefore, of special importance. Thus, the present study provides information that could be used in future studies that aim to estimate the prevalence of *Toxoplasma gondii* in wild mammals from southern Italy (Campania region) to better understand the parasite’s life cycle, its transmission dynamics, and the risk to public health [10], and to provide useful information for wildlife management and public health protection. We selected the Campania region due to its high human density and the proximity between humans and wildlife, exacerbated by many free-ranging domestic cats, which highly increases the risk of zoonotic transmission of this pathogen, as well as others (e.g., [11]).

In general, the host’s trophic level may influence exposure risk since top predators of endothermic vertebrates will be exposed to increased infection risks through the tissue-cyst transmission route [12]. In our study, we covered all mammal trophic levels from top predators to prey through mesocarnivores. Specifically, we tested the hypothesis that species-specific ecological traits related to trophic levels will lead to different exposure rates to *Toxoplasma gondii* through oocysts and tissue cysts. We expected a higher prevalence in top predators, intermediate in mesocarnivores, and lowest in mammal prey.

While it is believed that sex does not affect the prevalence of the parasite [13], age may affect the presence of *Toxoplasma gondii* in wild animals, as older animals are more likely to be infected [14,15,16]. Therefore, we also hypothesized that the prevalence of *Toxoplasma gondii* will change according to age, suggesting that the parasitic prevalence will be higher in adults, intermediate in sub-adults, and lower in juveniles. Lastly, the infection rate could be also related to the different geographic sampling areas.

In southern Italy, oocysts are expected to be more common in peri-urban areas where free-ranging domestic cats (*Felis catus*), both stray cats and pets, are allowed outdoors, acting as the most relevant definitive hosts of *Toxoplasma gondii* [17]. Most domestic cats found in urban areas are mainly kept indoors, apart from the limited free-ranging cat colonies protected by Italian national legislation [18]. However, free-ranging cats are widespread in rural and especially peri-urban areas of southern Italy, setting the scene for more frequent contact with wild mammals. In this context, we, therefore, hypothesized that wild mammals will encounter oocysts more frequently in areas where cats are freely moving, i.e., peri-urban sites, where a higher rate of *Toxoplasma gondii* is expected.

## 2. Materials and Methods

### 2.1. Study Area and Sample Collection

We sampled five mammal species between January 2020 and November 2022, from different areas of the Campania region (southern Italy). The region is mainly hilly and extends from 0 to 1890 m above sea level. The climate is Mediterranean with dry summers and rainy winters (e.g., [19]).

To test our hypotheses, we considered a total of 211 individuals as follows: wolves (*Canis lupus n* = 14), foxes (*Vulpes vulpes n* = 71), badgers (*Meles meles n* = 22), and roe deer (*Capreolus capreolus n* = 14), all found dead, while wild boars (*Sus scrofa n* = 90) were either found dead or killed by hunters. Additionally, we analysed stone martens (*Martes martes n* = 3), porcupines (*Hystrix cristata n* = 4), and otters (*Lutra lutra n* = 5), whose sample sizes were too small for statistical analysis.

Since all procedures followed the Italian and EU legislation, as part of the Regional Plans for Wildlife Surveillance [20], no approval from the ethical committees was needed. All individuals underwent a necropsy on the premises of the Istituto Zooprofilattico Sperimentale del Mezzogiorno (Portici, southern Italy), by professional staff (veterinarians and laboratory technicians) in a necropsy room. The organs that were collected for subsequent analysis depended on the animal’s condition. In general, if possible, brains, hearts, and/or muscles were removed with sterile scalpels, dissected, split out in sterile tubes, delivered within 24 h to the laboratory for biotechnological investigation and stored at −20 °C before DNA extraction.

### 2.2. Nucleic Acid Extraction

Twenty-five mg of each organ were minced with a sterile blade and transferred to a sterile Eppendorf containing 1 mL of pyrophosphate-buffered saline (PBS) solution. Samples were homogenized by TissueLyser (Qiagen) with one stainless steel bead for 3 min and centrifuged at 13,000 rpm for 3 min.

Nucleic acids were extracted from 200 μL of the homogenized samples using the MagMaxTM Viral/Pathogen II Nucleic Acid Isolation Kit (Applied Biosystems, Waltham, MA, USA), following the manufacturer’s instructions. Extracted nucleic acids were eluted in 80 μL elution buffer and immediately analysed by real-time PCR or stored at −20 °C until further processing. PCR inhibitors likely present in the samples were monitored by adding an external process control (EPC), namely murine norovirus [21], 5 µL of which (107 PFUmL^−1^) was spiked in each sample prior to extraction. EPC was amplified separately before testing for Toxoplasma gondii in each sample by real-time PCR with the following primers: MNoV F 5′-CACGCCACCGATCTGTTCTG-3′ and 5′-GCGCTGCGCCATCACTC-3′; and probe FAM-CGCTTTGGAACAATG-MGB-NFQ with the thermal profile indicated in the literature [22]. Results were analysed as already described [23].

### 2.3. Molecular Analysis

A real-time PCR was used to detect a small part of the *Toxoplasma gondii* B1 gene, as described by Sgroi et al. (2020). Briefly, 5 μL of template DNA was added to a reaction mixture in a final reaction volume of 25 μL containing PCR universal mastermix 1X, 0.5 μM of each primer (forward primer TOXO-F’ 5′-TCCCCTCTGCTGGCGAAAAG′-3′ and reverse primer TOXO-R’ 5′-AGCGTTCGTGGTCAACTATCGATT′-3′) and 0.2 μM of TaqMan probe (2 µM, 6FAM-TCTGTGCAACTTTGGTGTATTCGCAG-TAMRA) [24]. The thermal profile included an initial activation at 95 °C for 15 min, followed by 45 PCR cycles of 95 °C for 15 s and 60 °C for 1 min. Nuclease-free water was included as a negative control and genomic DNA from *Toxoplasma gondii* was obtained from the America Type Culture Collection (ATCC 50174D LGC Standards Italy) used as reference control. The amplifications were performed on a QuantStudio 5 real-time PCR system (Applied Biosystems, Foster City, CA, USA) thermal cycler.

### 2.4. Data Classification

To investigate possible spatial variation in *Toxoplasma gondii* prevalence among wild mammals, the analysed subjects were categorized according to the species’ trophic level as top predators (wolves), mesopredators (foxes and badgers), or prey (wild boars and roe deer). Additionally, we classified them by sex (females or males) and by age: juveniles (<1 year), sub-adults (1–2 years), and adults (>2 years) [25,26,27]. Age assessment was carried out empirically by comparing the biometric values of each individual with those known for the different species. The areas in which we collected animals were divided into three geographic zones: urban, peri-urban, or rural. Urban areas were characterised by human settlements with a high density of infrastructures and built environments, while areas considered rural occurred outside towns and cities, were dominated by farmland, and had very low occurrence or complete absence of buildings. We classified peri-urban areas following the UNESCO classification, according to which peri-urban areas are zones of transition from rural to urban land uses located between the outer limits of urban and rural environments (https://en.unesco.org/events/peri-urban-landscapes-water-food-and-environmental-security, accessed on 10 October 2022). The spatial distribution of prevalences was investigated by building a map with locations of sampling obtained via ArcGIS (version 10.3; ESRI, Redlands, CA, USA).

### 2.5. Statistical Analysis

Only wolves, foxes, badgers, wild boars, and roe deer had sufficiently large (>10) sample sizes for statistical analysis. The remaining species were not included in the analysis due to insufficient sample size, and prevalence data for them are provided in Supplementary Tables S1 and S2.

Contingency tables were analysed to assess the differences in prevalence associated with age, sex, sampling area, and organ infection prevalence. A *p*-value < 0.05 was considered statistically significant. The Fisher’s exact test was used and confidence intervals (CI) at 95% were also calculated. All statistical analyses were carried out with the jamovi software tool (http://www.jamovi.org/, accessed on 10 October 2022).

## 3. Results

A total prevalence of 21.8% (*n* = 46 positive samples out of 211) for *Toxoplasma gondii* was obtained from molecular analysis of the samples collected. According to species, the prevalence was as follows: wolf (*n* = 4/14; 28.6%, CI: 11.7–54.6), badger (*n* = 6/22; 27.3%, CI: 13.1–48.1), fox (*n* = 17/71; 23.9%, CI: 15.8–36.3), wild boar (*n* = 17/90; 18.9%, CI: 12.1–28.2), and roe deer (*n* = 2/14; 14.3%, CI: 4.0–39.9). No statistically significant difference in prevalence was found among species (Table 1).

**Figure 1:**
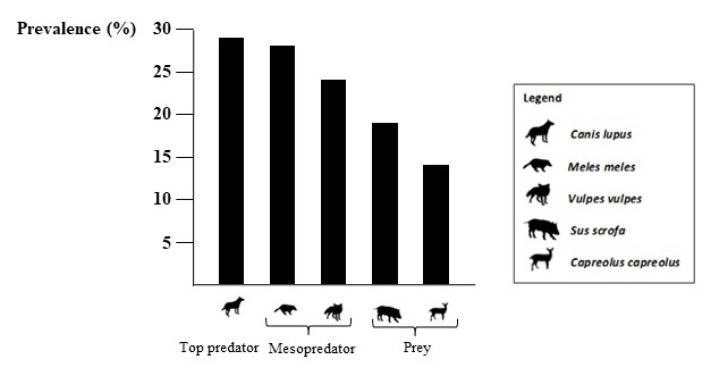
Molecular prevalence of *Toxoplasma gondii* in different synanthropic wildlife species of southern Italy, 2020–2022.

From a visual assessment of data distribution, we detected a decreasing trend (Figure 1) from apex predators (*n* = 4/14; 28.6%, CI: 11.7–54.6) to mesopredators (*n* = 23/93; 24.7%, CI: 17.1–34.4), and prey mammals (*n* = 19/104; 18.2%, CI: 12.0–26.8), but the pairwise comparisons of the prevalence between the groups was not significant (Table 1).

**Figure 2:**
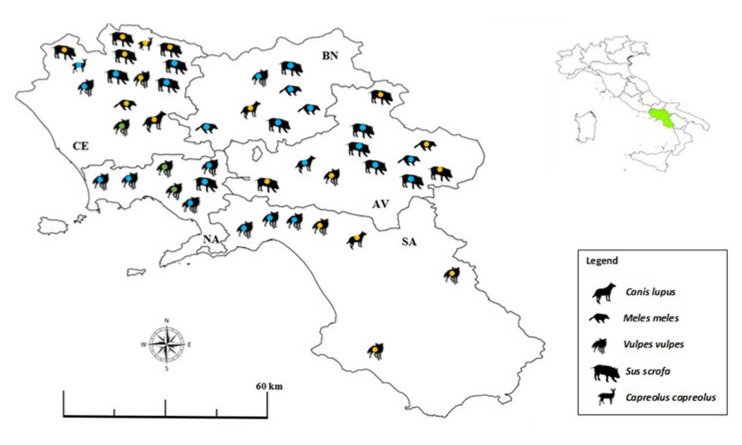
Map showing the distribution of synanthropic mammals (*n* = 46) that tested positive for *Toxoplasma gondii* DNA in different provinces (i.e., AV, Avellino, BN, Benevento, CE, Caserta, NA, Napoli, SA, Salerno) of the Campania region, southern Italy, in 2020–2022. Green = urban area; blue = peri-urban area; yellow = rural area.

We found individuals positive to *Toxoplasma gondii* in all provinces of the Campania region (Figure 2), with no statistical difference between provinces (χ^2^ = 0.73; *p* = 0.13).

Age and sex did not influence positivity in any of the species analysed. Only a boarder significance in the sex was associated with prevalence in the wild boars (*p* = 0.053), where positivity was more frequently associated with males (Table 2).

For wolves, land use comparisons were only possible between peri-urban and rural areas because no individual was found in urban areas (Table 2). Although the sample size was small, wolves from rural areas were more frequently positive than those from peri-urban areas. Instead, wild boars found in peri-urban areas were more frequently positive than those from rural and urban areas, respectively (Table 2).

Out of the 465 organs analysed, the highest prevalence rate was found in the heart (25/163, 15.3%), followed by the brain (12/112, 10.7%) and the muscle (18/190, 9.2%), but such differences were not statistically significant (Table 3).

## 4. Discussion

Our survey revealed a high prevalence of *Toxoplasma gondii* (21.8%) in wild mammals found in different territories of the Campania region (southern Italy), i.e., 1 out of 4.4 synanthropic wild mammals harboured the parasite DNA. This finding confirmed the pattern known from other European regions. For instance, Calero-Bernal et al. (2015) found an overall prevalence of *T. gondii* infection of 32.2% in 183 wild mammals from southwestern Spain belonging to six different species, three of which (foxes, wild boars, and roe deer) were in common with the present study [28].

We also found that although mammals at higher trophic levels exhibited a higher prevalence of *Toxoplasma gondii*, no statistically significant differences were noticed between the categories. Ferroglio et al. (2014) [29] showed a higher prevalence in carnivorous and omnivorous wild mammals (red fox and wild boar) than in herbivores (red deer and chamois), which was interpreted because of the cumulative effect of the parasite’s predator–prey cycle (see also [30]). This would link to the higher probability of a carnivorous or omnivorous species ingesting infected tissues vs. that of a herbivore, which may only ingest oocyst-contaminated food plants, soil, and/or drinking water [12]. The different result herein obtained was probably associated with the high degree of anthropization typical of the Campania region, and the increasingly synanthropic habits of the mammal species we considered [24,31,32,33]. All factors increased the likelihood of pathogen-spread in areas where free-ranging domestic cats were abundant and their direct or indirect contacts with wildlife frequent. Additionally, all species considered in this study, except the roe deer, were carnivorous or omnivorous [34,35,36], which may have partially masked the predicted pattern, weakening its statistical significance (while the visual trend was retained).

The high circulation of the parasite among syntropic mammal species we found, besides being characterized by the above-mentioned lack of differences across trophic levels, was also confirmed by the absence of statistically significant differences in overall prevalence among provinces of the study region.

Like previous work (e.g., [13,37]), we found no difference among animal sex except a boarder significance (*p* = 0.053) in the wild boars, with males more often positive than females. A possible explanation for this is that males tend to more frequently be in contact with potential sources of infection, since they typically show larger home ranges than females [38]. Moreover, wild boar males are typically solitary and more risk-prone than females (living in groups along with their young), and so, males may tend to use food-rich, anthropised habitats more than females [39]; all the more in areas such as the one we considered, where wild boar is routinely hunted. In addition, solitary males can more easily feed on the carcasses of animals injured in hunting (e.g., wild boars and foxes) by assuming cysts with bradyzoites and/or by carrying out a scavenging action [39]. In human-altered habitats, contact with cat-driven sources of infection is much more likely, which would explain the sex-biased prevalence we recorded [32]. Wild boars were recently found to be attracted by cat food in anthropized areas, a concerning situation in terms of domestic cat-to-wild boar transmission of pathogens, especially *Toxoplasma gondii* [32], and solitary individuals such as males were more likely to obtain access to this kind of food source without being noticed and driven out.

Unlike previous work [13], we found no difference among age classes in the rates of positivity likely due to the limited sample sizes we considered, but also to the high circulation of the parasite in the environment, nullifying age-biased patterns. In general, older animals were more likely to have exposure with the parasite than younger ones, having a higher probability of being infected [13], but this general pattern is likely to change considerably under different environmental and epidemiological conditions.

Land use showed limited influence over positivity across the species considered, probably as a result of the high circulation of free-ranging cats, and as a consequence of the protozoan, in the study area. A borderline significance was observed in wolves, suggesting that in rural areas, these would be more affected than in peri-urban environments. Wolves in rural environments may have high probability to be in contact with *Toxoplasma*-infected prey, such as wild boars, the wolf’s main prey in the Italian Apennines (e.g., [40]). However, this result must be taken cautiously due to the very small sample size available and the typically large home ranges shown by resident wolves, spanning over territories well over 100 km^2^ across many habitat types, or even more dispersing individuals, moving over up to thousands of km [41]. Classifying such highly opportunistic and mobile predators according to the land use of the area where they died may have little ecological meaning. The other species that showed a land use-biased positivity, the wild boar, had a clearer pattern, with subjects dwelling in peri-urban areas more frequently positive than those in urban or rural sites. This was probably explained in terms of the higher likelihood of contact with free-ranging cats typical of such environments, as discussed above.

We also found, as in previous studies [42], that the tropism of infection herein observed was equal for all organs considered. This record is in agreement with previous surveys by Alves et al. (2019), who observed the viability of *Toxoplasma gondii* cysts in organs of pigs experimentally infected with 3 × 10^3^ oocysts of a *Toxoplasma gondii* isolate, demonstrating that the parasite can form tissue cysts in all organs [43].

This study revealed a high circulation of *Toxoplasma gondii* in wild mammals of southern Italy, which may represent public health concern due to the virulence of the parasite in humans. The prevalence found in wild boar samples, especially muscles, highlights the relevance of these findings for public health since this species is commonly used to prepare traditional raw meat products, potentially representing an important source of *Toxoplasma gondii* infection to consumers. Wild boar is likely to play a major role in human infection as well as in the epidemiological cycle of *Toxoplasma gondii* infection [44].

The consumption of raw meat products from wild boar muscles also brings about a risk of other zoonotic pathogen infections [45]. Once more, these results highlight the urgent need to implement surveillance of zoonotic pathogens in wildlife, especially those related to human consumption.

The incidence of acute human toxoplasmosis hospitalizations in the Campania region is of 0.72/per 100,000 inhabitants, as reported by hospital discharge records [46]. Most patients were aged less than 1 year of age, followed by adults between 25 and 44 years of age with no gender difference [46]. Health problems related to toxoplasmosis are, however, highly underestimated, as the parasite infection is linked to many chronic diseases [47,48].

Free-roaming domestic cats play an important role in spreading toxoplasmosis with marked impacts on biodiversity and human health. A science-based approach for better management of free-roaming cats is required from a broader political and legislative perspective, regulated by the consensus in the animal ecology, conservation, and welfare communities [49]. In Italy, urban free-roaming cats living in colonies are protected by the law (no. 281/1991), namely: (i) cats have the recognized right to live free; (ii) neutering of cats by the Veterinary Services of the Local Health Unit is compulsory; (iii) cat caretakers are institutionalized [18]. Although this degree of protection has certainly improved the conditions of stray cats, its outcome in terms of consequences for urban animal biodiversity as well as human health is still potentially concerning. We propose these colonies be systematically monitored for their effects on wildlife and the possible zoonotic consequences assessed to develop appropriate management strategies [11]. Our findings highlighted the need to establish surveillance programs and preventive strategies in a multidisciplinary one health approach to the monitoring of wildlife species, to protect biodiversity and mitigate the risk of zoonotic transmission to humans. Accordingly, more efforts by the health stakeholders are required for the game meat inspection, such as for wild boar and roe deer foodstuffs, in order to prevent *Toxoplasma gondii* infections to consumers.

## Figures and Tables

**Table 1:** Significance levels of Fisher’s exact test in pairwise comparisons of *Toxoplasma gondii* prevalence between mammal species and trophic levels.

Species	*p*
Wolf vs. fox	0.740
Wolf vs. wild boar	0.474
Wolf vs. roe deer	0.648
Wolf vs. badger	1.000
Fox vs. wild boar	0.444
Fox vs. roe deer	0.726
Badger vs. fox	0.781
Wild boar vs. roe deer	1.000
Badger vs. wild boar	0.388
Badger vs. roe deer	0.441
Top predators vs. mesopredators	0.748
Top predators vs. mammal prey	0.470
Mesopredator vs. mammal prey	0.299

**Table 2:** Prevalence of *Toxoplasma gondii* DNA in synanthropic mammal species of southern Italy according to sex, age, and dominant land use in 2020–2022. Missing categories (indicated with “-“) refer to the absence of samples.

Variable	Species				
	Wolf Pos/Tot (%)	Fox Pos/Tot (%)	Wild Boar Pos/Tot (%)	Roe Deer Pos/Tot (%)	Badger Pos/Tot (%)
Sex					
Male	2/5 (40.0)	9/39 (23.1)	11/37 (29.7)	2/12 (16.7)	5/15 (33.3)
Female	2/9 (22.2)	8/32 (25.0)	6/53 (11.3)	0/2 (0)	1/7 (14.3)
	*p* = 0.580	*p* = 1.000	*p* = 0.053	*p* = 1.000	*p* = 0.616
Age					
Juvenile	-	3/15 (20.0)	1/10 (10.0)	-	1/2 (50.0)
Sub-adult	0/2 (0)	3/15 (20.0)	10/52 (19.2)	0/5 (0)	1/5 (20.0)
Adult	4/12 (33.3)	11/41 (26.8)	6/28 (21.4)	2/9 (22.2)	4/15 (26.7)
	*p* = 1.000	*p* = 0.866	*p* = 0.858	*p* = 1.000	*p* = 0.799
Dominant land use					
Urban	-	3/16 (18.7)	0/7 (-)	-	0/1 (-)
Peri-urban	1/10 (10)	9/40 (22.5)	10/26 (38.5)	1/10 (10.0)	4/15 (26.7)
Rural	3/4 (75.0)	5/15 (33.3)	7/57 (12.3)	1/4 (25.0)	2/6 (33.3)
	*p =* 0.040	*p* = 0.695	*p* = 0.010	*p* = 0.505	*p* = 1.000

**Table 3:** Prevalence of *Toxoplasma gondii* DNA in synanthropic mammal species of southern Italy in 2020–2022 according to the organs examined.

Species	*p*-Value	Organs		
		Heart Pos/Tot (%)	Brain Pos/Tot (%)	Muscle Pos/Tot (%)
Wolf	*p* = 0.822	3/11 (27.3)	1/11 (9.1)	1/7 (14.3)
Fox	*p* = 0.485	8/48 (16.7)	4/48 (8.3)	6/61 (9.8)
Wild boar	*p* = 0.580	10/82 (12.2)	5/37 (13.5)	7/86 (8.1)
Roe deer	*p* = 0.405	1/4 (25.0)	Not performed	1/14 (7.1)
Badger	*p* = 0.100	3/18 (16.7)	2/16 (12.5)	3/22 (13.6)
Total	*p* = 0.228	25/163 (15.3)	12/112 (10.7)	18/190 (9.5)

## Data Availability

Not applicable.

## Supplementary Materials

The following supporting information can be downloaded at: https://www.mdpi.com/article/10.3390/pathogens12030471/s1, Table S1: Prevalence of *Toxoplasma gondii* DNA in synanthropic wildlife species of southern Italy according to sex, age, and area, 2020–2022. Missing categories (indicated with “-“) refer to the absence of samples; Table S2: Prevalence of *Toxoplasma gondii* DNA in synanthropic wildlife species of southern Italy according to the organs examined, 2020–2022. Missing categories (indicated with “-“) refer to the absence of samples.
