# Supplementary material for: Molecular Survey of Toxoplasma gondii in Wild Mammals of Southern Italy
Source: Pathogens. 2023 Mar 16;12(3):471. doi: 10.3390/pathogens12030471 (PMC10051445; doi:10.3390/pathogens12030471)
Supplement: Supplementary file 1 [file pathogens-12-00471-s001.zip › pathogens-2271888-supplementary.pdf]

Table S1. Prevalence of *Toxoplasma gondii* DNA in synanthropic wildlife species of southern Italy according to sex, age, and area, 2020-2022. Missing categories (indicated with "-") refer to the absence of samples.

**Variables**	**Species**		
	**Porcupine**<br>**Pos/Tot (%)**	**Otter**<br>**Pos/Tot (%)**	**Stone marten**<br>**Pos/Tot (%)**
**Sex**			
Male	1/2 (50.0)	1/3 (33.3)	1/2 (50.0)
Female	1/2 (50.0)	1/2 (50.0)	0/1 (0)
**Age**			
Juvenile	1/1 (100)	1/2 (50.0)	(-)
Sub-adult	(-)	(-)	(-)
Adult	1/3 (33.3)	1/3 (33.3)	1/3 (33.3)
**Area**			
Urban	(-)	(-)	1/1 (100)
Peri-urban	1/1 (100)	0/1 (0)	0/2 (0)
Rural	1/3 (33.3)	2/4 (50.0)	(-)

Table S2. Prevalence of *Toxoplasma gondii* DNA in synanthropic wildlife species of southern Italy according to the organs examined, 2020-2022. Missing categories (indicated with "-") refer to the absence of samples.

**Species**	**Organs**		
	**Heart**<br>**Pos/Tot (%)**	**Brain**<br>**Pos/Tot (%)**	**Muscle**<br>**Pos/Tot (%)**
**Porcupine**	1/2 (50.0)	0/2 (0)	1/4 (25.0)
**Otter**	1/5 (20.0)	1/2 (50.0)	(-)
**Beech marten**	1/3 (33.3)	1/3 (33.3)	0/3 (0)
